# Microfluidic Blood Separation: Key Technologies and Critical Figures of Merit

**DOI:** 10.3390/mi14112117

**Published:** 2023-11-18

**Authors:** Karina Torres-Castro, Katherine Acuña-Umaña, Leonardo Lesser-Rojas, Darwin R. Reyes

**Affiliations:** 1 Biophysical and Biomedical Measurements Group, National Institute of Standards and Technology (NIST), 100 Bureau Drive, Gaithersburg, MD 20899, USA; karina.torrescastro@nist.gov; 2 Theiss Research, La Jolla, CA 92037, USA; 3 Medical Devices Master’s Program, Instituto Tecnológico de Costa Rica (ITCR), Cartago 30101, Costa Rica; 4 Research Center in Atomic, Nuclear and Molecular Sciences (CICANUM), San José 11501, Costa Rica; leonardo.lesser@ucr.ac.cr; 5 School of Physics, Universidad de Costa Rica (UCR), San José 11501, Costa Rica

**Keywords:** blood contents, blood sorting, separation of blood, figures of merit, lab-on-a-chip, microfluidic separations, pathogenic bacteria, red blood cells

## Abstract

Blood is a complex sample comprised mostly of plasma, red blood cells (RBCs), and other cells whose concentrations correlate to physiological or pathological health conditions. There are also many blood-circulating biomarkers, such as circulating tumor cells (CTCs) and various pathogens, that can be used as measurands to diagnose certain diseases. Microfluidic devices are attractive analytical tools for separating blood components in point-of-care (POC) applications. These platforms have the potential advantage of, among other features, being compact and portable. These features can eventually be exploited in clinics and rapid tests performed in households and low-income scenarios. Microfluidic systems have the added benefit of only needing small volumes of blood drawn from patients (from nanoliters to milliliters) while integrating (within the devices) the steps required before detecting analytes. Hence, these systems will reduce the associated costs of purifying blood components of interest (e.g., specific groups of cells or blood biomarkers) for studying and quantifying collected blood fractions. The microfluidic blood separation field has grown since the 2000s, and important advances have been reported in the last few years. Nonetheless, real POC microfluidic blood separation platforms are still elusive. A widespread consensus on what key figures of merit should be reported to assess the quality and yield of these platforms has not been achieved. Knowing what parameters should be reported for microfluidic blood separations will help achieve that consensus and establish a clear road map to promote further commercialization of these devices and attain real POC applications. This review provides an overview of the separation techniques currently used to separate blood components for higher throughput separations (number of cells or particles per minute). We present a summary of the critical parameters that should be considered when designing such devices and the figures of merit that should be explicitly reported when presenting a device’s separation capabilities. Ultimately, reporting the relevant figures of merit will benefit this growing community and help pave the road toward commercialization of these microfluidic systems.

## 1. Introduction

The development of new approaches and technologies to separate blood components has gained traction in the past few decades. Numerous health conditions have been studied using those new approaches, and these technological advances have contributed to the development of new treatments and theranostics for diseases such as cancer and neurodegenerative and infectious diseases [1,2].

**Figure 1:**
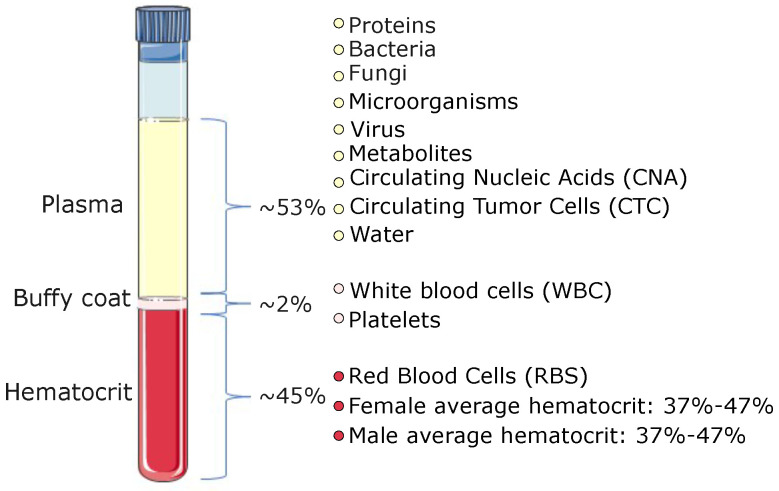
Human blood composition (figure adapted from [18]).

Blood samples containing circulating biomarkers are powerful indicators of the presence and evolution of a disease [3,4]. However, the separation of the components in blood samples is challenging due to their complex matrix composition. As shown in Figure 1, human blood is composed of heterogeneous cell subpopulations. Nevertheless, cancer can be tracked in blood by detecting circulating tumor cells (CTCs), cancer cells [5], and other biomarkers. In the same way, bacterial infections [6,7] and some parasites (e.g., malaria) [8] can be detected and correlated to an increased risk of death, anemia, and infection recrudescence [9]. Some viruses such as hepatitis E [10], hepatitis C [11], human immunodeficiency virus (HIV) [12], and others can be detected and diagnosed from human blood samples as well [13]. An example of this has been reported recently in the form of a microfluidic enzyme-linked immunosorbent assay (ELISA) chip for detecting COVID-19 antibodies using blood plasma [14,15].

Traditionally, macro blood separations in clinical and biology lab settings require multiple preparation steps, including filtering and centrifugation. Thus, large volumes of blood samples are required from patients [16]. Furthermore, these processes are time-consuming and prone to cross-contamination and low analyte purity. Abnormal concentration levels of different blood subpopulations are linked to different health conditions. For example, a low concentration of RBCs (or erythrocytes) is linked to different types of anemia; high concentrations of thrombocytes are linked to blood clotting; low levels (concentrations) of T and B lymphocytes are linked to diseases such as HIV, pneumonia, hepatitis, Hodgkin’s disease, and aplastic anemia; and high concentrations of monocytes are linked to the presence of syphilis and tuberculosis. Moreover, high levels of neutrophils are linked to bacterial infections, some viral infections, sepsis, chronic and acute inflammation, etc. Thus, obtaining these blood subpopulations gives important insights and can shed light on patients’ health conditions [17].

Microfluidic separations are an attractive approach to obtaining high-quality samples since the characteristic flow of these micro-scale systems allows tracking bioparticles in a deterministic manner [19]. This characteristic flow feature, coupled with clever device design and external forces, enables the separation of blood sample subpopulations of interest with high cell throughput (cells per minute), efficiency, and purity (>80%), at a lower cost and with small sample volumes in the range from microliters to nanoliters [20,21]. Ideally, more integrated devices that separate different blood components and perform other operations in the same device, such as quantification of the separated fractions and metabolomics, proteomics, and transcriptomics analysis, are desired [22,23,24]. These highly integrated devices will save a significant amount of time and resources (e.g., reagents) and can also improve the recovery or collection yield of the separated samples [25,26]. For instance, a paper-based point-of-care (POC) aptasensor using gold nanoparticles coupled with colorimetric analysis for monitoring potassium ion (K+) concentrations using whole blood has been reported. This method can detect potassium ions within a concentration range from 0.05 × 10^−3^ mol/L to 9 × 10^−3^ mol/L and have a limit of detection (LOD) of 0.01 × 10^−3^ mol/L. This device demonstrates that low-cost POC microfluidic sensors with on-chip blood separations (serum from whole blood) are attainable [27]. Another example of the capability of these devices is in the separation of extracellular vesicles such as exosomes. Exosomes are essential in cell communication and can be used as biomarkers for Parkinson’s and Alzheimer’s disease and diabetes, to name a few. Thus, the purification of exosomes from whole blood is highly desired for studying these pathologies. Microfluidic devices that use external forces to purify exosomes from blood using techniques such as magnetophoresis for diagnosing pancreatic cancer [28] and dielectrophoresis to separate red blood cells (RBCs) from whole blood to detect prostate-specific antigen (PSA) [29] in the same chip have been reported. Overall, microfluidic devices for blood separation and quantification of the analytes in blood are promising tools for POC diagnostics [30].

To design and understand how these microfluidic chips operate, we turn to the Navier-Stokes equation, which describes fluid flow systems [31] by solving Newton’s second law of motion for fluids. Due to the characteristic dimensions of the microchannel networks found in lab-on-a-chip (LoC) [32] and organ-on-a-chip (OoC) [33] devices, surface forces become more significant than volumetric forces. Therefore, we must adjust our fluidic perception when dealing with these types of microscale devices. A relevant aspect of microfluidic systems is that we can trace fluid flow streamlines and particles such as RBCs back to their feeding origin. This is possible due to the characteristic laminar flow regime found in these systems. To understand the physical phenomena that describe flow motion in these micro-systems, the Navier-Stokes equation, which constitutes the system’s conservation of momentum and the continuity equation [34] for mass conservation, must be solved to understand the fluid dynamics inside a microfluidic channel. With this, we can establish relations between the microfluidic system pressure and flow velocity and track the movement of particles in the channel at all times. When an external force is applied to separate different blood components, the exerted force on the particles (e.g., cells) will be expressed in terms of the resulting force experienced by the particles based on their properties, volume, and interaction with the applied force. The resulting main forces for separating particles will be briefly discussed in Section 4.

We will briefly go over some passive microfluidic devices that rely on the interaction of hydrodynamic forces with different channel geometries and active devices used for continuous blood (dynamic) separations, which present novel designs or distinct device features that enable high-throughput (number of cells per min) blood separations [35]. Moreover, this review discusses critical figures of merit that should be reported to compare microfluidic blood separation devices that apply different microfluidic separation techniques. For the purpose of this review, we will refer to active techniques as the methods that require the application of external forces to separate blood components. These external forces can be of electric, magnetic, or acoustic nature. We will provide an overview of the active techniques’ state-of-the-art perspective on how the field is moving toward more efficient separations and the main parameters that should be considered for designing active microfluidic devices. Reporting key figures of merit will help with scaling up the production of these microfluidic blood separation devices to accelerate their commercialization.

In recent years, there have been interesting reviews of passive separation methods that cover a myriad of microfluidic techniques and applications for separating different types of bioparticles [36,37,38,39]. This review by no means intends to cover passive microfluidic separation theory or methods but instead encourages the reader to consider what metrics they should report when proposing a new microfluidic separation device, irrespective of which microfluidic separation technique they used. Devices can separate blood components into passive, active (applied external forces), or a combination of different techniques. To compare which methods are more successful in separating blood components, or any bioparticle for that matter, we need and encourage authors to report a set of metrics for their devices so that everyone can assess which devices are better equipped for separating the blood components of interest.

## 2. Key Device Performance Figures of Merit: Definitions

It is important to establish a framework for assessing the quality of microfluidic separations achieved by the different devices that are currently developed for separating blood and its components. The following figures of merit are not new to the microfluidic community and are currently used to report the device’s achievements, but more often than not, these key figures of merit are arbitrarily selected by authors, and some are chosen to be explicitly reported in publications whereas some others are neglected. Despite the fact that there is not an established standard for this community to report the following figures of merit, we believe this field will benefit if more researchers report all the figures of merit we refer to in this article when presenting their device’s separation yields. Reporting these results will help make decisions on what type of strategies can be used for separating blood components and will help to establish a microfluidic blood separation roadmap that will promote the development and commercialization of these devices.

The following are key figures of merit for microfluidic blood separations that, in our estimation, can provide a more complete frame of reference for a reader when attempting to compare different techniques. These figures of merit are the sample initial and output concentrations, separation efficiency, purity, flow rate, and throughput.

### 2.1. Input and Output Concentrations

The input concentration (cells per milliliter) tells us if the sample to be separated is diluted blood or whole blood. This parameter is important to assess the sample complexity and its potential use in clinical settings based on the number of steps that must be taken before injecting the sample into the device for separation. The output concentration is the final concentration of the cell or bioparticle of interest after the separation stage in a microchannel. The output concentration is a metric used to assess the enrichment or sample concentration with respect to the initial sample input concentration.

### 2.2. Efficiency

The separation efficiency is defined as the ratio between the separated target cells in the device output and the total input sample count [40]. The separation efficiency is usually reported as a percentage (%).

### 2.3. Purity

Purity is a selectivity measurement that indicates the number of particles of interest (desired) the separated fractions have with respect to the sample’s total number of particles. Purity is also reported as a percentage (%).

### 2.4. Flow Rate and Throughput

The flow rate (microliters per second or milliliters per minute) is a metric that helps predict how long it will take to separate samples in a device. It also provides an idea of how much of an input sample is required (volume in milliliters). The throughput (cells per minute), on the other hand, is the parameter for assessing how many cells or particles are separated over time.

## 3. Passive Techniques

Hydrodynamic microfluidic separation techniques exploit the properties of laminar flow and the interaction of flow with bioparticles to ultimately help design microdevices capable of separating bioparticles for biomedical and other applications [41,42]. Hydrodynamic separation principles such as pinched flow fractionation, hydrophoresis, and gravitational methods [43,44] are discussed extensively elsewhere [45,46]. Therefore, our focus will mainly be on dynamic application (continuous flow) separations that enable high-throughput blood separations, such as inertial microfluidics and deterministic lateral displacement techniques.

Inertial microfluidic methods have become a widely used hydrodynamic approach for separating particles due to their high throughput capability and simplicity of use compared with other passive techniques. These devices exploit the channel geometries [47] and lift forces experienced by particles close to a Reynolds number equal to one, thus affecting the particle’s equilibrium positions. This particle displacement toward an equilibrium position is known as inertial particle migration. This migration allows the particles to be separated and collected downstream without using additional external forces (F_ext_) [48,49].

Deterministic lateral displacement (DLD) is a microfluidic technique used to separate particles based on their size, deformability, electrical properties, and interaction with a series of pillars designed specifically for displacing particles diagonally (displacement mode) when their size is above a designed cut-off particle diameter. In contrast, when they are smaller than the cut-off diameter, also known as the critical diameter (D_c_), they follow the fluid flow streamlines in what is typically called zigzag mode. DLD has the benefit of being a passive separation technique with a high resolution. DLD devices are particularly attractive for healthcare applications (e.g., isolating different blood contents, purification, enrichment, and fractionation of different blood-circulating biomarkers from samples such as whole blood) [50,51]. Inertial microfluidics and DLD arrays have been used in hybrid or integrated devices as a separation stage prior to active separations that use different external forces. Hybrid devices with measuring or cell-counting sections allow quantifying the separated fractions through traditional optical or fluorescence cytometry [52,53] and electrical or impedance cytometry [54,55]. More recently, acoustic flow cytometers (AFCs) have been developed to count RBCs and white blood cells (WBCs) [56] and detect bacteria in blood [57].

## 4. Passive Blood Separations

### 4.1. Passive Separation of Blood Cells

We will cover applications that span from separating plasma from blood to the separation of other components of blood including, among others, RBCs, WBCs [58], platelets, biomarkers (e.g., CTCs) [59], and dendritic cells [60]. Different channel configurations have been explored for the separation of blood components, including straight channels, serpentine channels with curved [61] or sharp angles, as well as divergent serpentine microchannels [62] and other serpentine geometry configurations [63], spiral channels [64], and expansion-contraction channels for high throughput hydrodynamic separations.

Xiang and Ni described in their inertial microfluidics review that particle separation is the most important function of spiral and expansion-contraction designs. On the other hand, curved and straight channels are mainly used for sample focusing [65]. Thus, we will explore spiral and expansion-contraction devices for separating blood contents. For a more in-depth overview of other inertial techniques as well as general hydrodynamic applications, please refer to the works of Wang et al. and Kim et al. [38,48].

**Figure 2:**
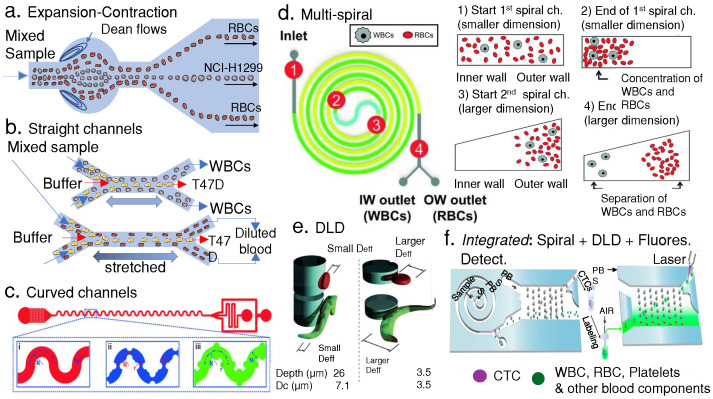
(**a**) Expansion-contraction channels for the separation of lung cancer cells (NCI-H1299) from RBCs (figure adapted from [67]). (**b**) A bimodal stretchable straight channel for separating breast cancer cells (T47D) from WBCs and T47D from RBCs (stretched) (figure adapted from [69]). (**c**) Sinusoidal (curved) inertial separation of T47D cancer cells, RBCs, and WBCs (reproduced from [76] with permission from the Royal Society of Chemistry). (**d**) Multi-spiral separation of WBCs and RBCs (reproduced from [71] with permission from the Royal Society of Chemistry). (**e**) Deterministic lateral displacement (DLD) separation of WBCs, RBCs, and blood parasites (reproduced from [73] with permission from the Royal Society of Chemistry). (**f**) Integrated device for CTC, WBC, and RBC separation from whole blood with fluorescence flow cytometry (reprinted with permission from [82], copyright (2019) American Chemical Society).

Hydrodynamic separation techniques are most effective when particles have significant size differences [66]. Particles can be collected downstream by increasing the spacing of the particle flow streamlines via channel expansions. Channel contractions and expansions have been used to separate plasma from blood and lung cancer cells from RBCs by inducing Dean flows in channel expansions (see Figure 2a). Dean flows, in turn, affect cell equilibrium positions due to lifting forces and their distinct center of mass. Liu et al. designed a hydrodynamic device to separate lung cancer cells (NCI-H1299) from RBCs with a series of expansions and contractions in a microfluidic channel. Figure 2 shows a schematic of one of their proposed geometries. They explored different Dean flow patterns to tune their microchannel expansions to separate cancer-derived cells (center) from RBCs (lateral) [67]. In addition, Mach et al. reported a cell throughput of 8 mL/min using a massive parallel device that first removed 80% of the pathogen *E. coli* and then enriched the RBC’s concentration by four times from a sample input with a volume fraction of 0.5% [68].

Particle migration across the channel width depends on the channel design (e.g., straight versus curved) and length. This geometry constraint prevents tuning the design for different types of samples since the channel is designed to induce specific equilibrium positions for the particles to be separated. Hence, if another sample presents significant cell size differences, then another device should be designed for that purpose. One exciting approach to address this issue is changing the channel dimensions dynamically by stretching them. Such changes in dimensions induce further changes in the equilibrium positions of the particles in the channel, thus allowing the separation of other samples with different size ranges. Fallahi et al. showed that by stretching a polydimethyl siloxane (PDMS) chip, it is possible to change the inertial migration of the particles to different equilibrium positions using the same device. As a result of changing the length of the microchannel, the separation size resolution changes as well. As a proof of concept, they separated WBCs from T47D (breast cancer) with the chip in its relaxed position and the same type of cells from RBCs in its stretched mode (Figure 2b). This is indeed an interesting concept since it opens the possibility of using the same chip design to separate cells with different cell size resolutions. Figure 2b shows a sketch of their stretchable device. They reported 97.4% recovery of T47D cells and a purity of 82.6% from WBCs and 98.6% recovery and a purity of 90% for T47D cancer cells from diluted whole blood [69].

Spiral channels use secondary Dean flows to create cell flow profiles along the channel width that branch downstream in different channel lanes to collect the separated cell streamlines. To design spiral channels for particle separations, the channel cross-section, Reynolds number (Re), and radius of the channel curvature must be taken into account to obtain the desired strength of the Dean flow in a curved channel characterized by the Dean number (De) relation. Other calculations must be carried out to define the channel lengths and expected cell equilibrium positions for separation that depend heavily on the cell size. However, other cell properties (density, morphology, deformability, rotation, and others) can also play a role, depending on the application [70]. Jeon et al. developed a spiral design to separate WBCs from RBCs with a changing microchannel profile with automated operation. They parallelized the spirals to improve their device throughput and reported up to >99.99% RBC removal, ≈80% WBC recovery, >85% WBC purity, and 12-fold concentrated WBCs compared with their input sample. The complete process takes less than 5 min with high reliability and repeatability. Notably, they developed a version of their platform that can be operated manually for lower-income settings, making it more user-friendly (see Figure 2d) [71]. Xiang et al. reported on a battery-driven stand-alone system for separating three different spiked tumor cells from diluted blood. This system incorporates a sheath flow to a spiral design, ultimately producing efficiencies higher than 90%, cell recoveries of more than 80%, and an enrichment fold of more than 2.5 times [44]. Other successful methods for enrichment of CTCs from blood buffy coats have also been reported when using spiral devices with cycles to separate or remove WBCs from a spiked sample to mimic diagnostic leukapheresis (removal of WBCs) [72]. In their paper, they reported removal of 99.98% of WBCs, showing their chip potential for enriching CTCs with lower amounts of WBCs.

As was mentioned before, DLD is a microfluidic hydrodynamic technique for separating particles mainly based on their size, deformability, and their interaction with a series of pillars. Those pillars are designed purposefully for displacing particles diagonally (displacement mode). When particles are smaller than the designed D_c_, they follow the flow streamlines (zigzag mode). DLD has the benefit of being a high-resolution, continuous flow separation technique. Thus, it is suitable for isolating, purifying, enriching, and separating blood and its contents for healthcare applications. DLD arrays can be integrated with other techniques on the same chip to perform different blood cell separations and sub-fractionate blood-circulating biomarkers [50,51].

Holm et al. designed a DLD device to separate WBCs and RBCs from a blood parasite (*T. cyclops*). They explored the DLD pillars’ depth effect and showed how RBCs, WBCs, and the blood parasite rotated due to shear forces (Figure 2e), thus allowing the separation of WBCs from RBCs and the parasite in the first stage. In the second stage, the parasites and the RBCs become displaced toward the device’s channel walls by decreasing the post array height to confine the parasites and RBCs to a flat position (maximum diameter). Finally, in the third stage, the parasite is separated by the displacement mode when the post array height is increased, while the RBCs follow the zigzag mode. Ultimately, both are collected downstream. The pillar configurations for the first and second stages are shown in Figure 2e [73]. An inertial microfluidic cube for extracting WBCs from whole blood was reported by Zhu et al. [74]. This system showed an efficiency of more than 80%, with WBCs having high viability with a WBC concentration for the collected samples of 2.3 × 10^5^ cells/mL and a sample input flow rate of 0.16 mL/min with a buffer input flow rate of 2.24 mL/min. Moreover, Torres-Castro et al. designed an integrated DLD device with impedance measurements on-chip to quantify the separated fractions of a specific type of WBCs (i.e., macrophages) based on the number of events detected in each collection outlet and correlated immune-activated vs. non-activated macrophage states with their phase shifts [75].

Inertial hybrid devices such as cascaded sinusoidal ones are high-throughput devices and produce high-purity separations. A device with curved, serpentine and different geometries for expansions and contractions was designed as a proof of concept for potential CTC separation (Figure 2c). The authors used this to separate cancer cells (T47D breast cancer cells) from the blood by creating different Dean flows. Curved channels with expansions and contractions in a cascaded device performed the separation in two stages to improve the cancer cells’ purity, with a throughput of 9 × 10^7^ cells/min (Figure 2c) [76]. Another device separated cancer cells (MCF7) from diluted blood with reported efficiencies of >94% and purities of 92% at a flow rate of 1 mL/min. This system uses a hybrid approach of inertial separation with downstream magnetophoresis. The device showed an increase of more than 5% and 8% in efficiency and purity, respectively, compared with the same device without applying magnetophoresis forces [77].

Other types of devices combine inertial effects with post arrays to separate and capture blood components. For instance, clog-free devices for separating CTCs from blood were designed with different tear-shaped microposts capable of operating at high flow rates (1 mL/min), with reported efficiencies of more than 80% [78]. These micropost shapes exploit rotation-induced lift forces in combination with particle densities that push larger particles further from the micropost wall. In these devices, RBCs bypass the microposts, while CTCs are captured downstream in the micropost array crevices. There are multiple examples of inertial separations of CTCs [79] and other cell types from blood. We invite the reader to investigate this topic further in recent reviews of inertial or passive separations for different bioparticles [65,80].

Continuous-flow hydrodynamic separation techniques are versatile not only for separating blood contents but also as an intermediate step that can be combined with other hydrodynamic techniques to separate more heterogeneous samples containing three or more types of blood cells (subpopulations) [81]. These continuous-flow hydrodynamic approaches can also be integrated with different external forces for active separations based on their contrast factors. Additionally, quantifying the separated fractions in the same device is highly desirable since it saves time. More importantly, it saves valuable samples by avoiding off-chip handling which leads to sample loss, making the process more expensive. For instance, Pei et al. integrated a spiral inertial separation with a DLD stage and quantified CTCs from blood with on-chip fluorescence flow cytometry, achieving 92% purity (see Figure 2f) [82].

Figure 2 below shows some of the passive separation devices already discussed.

### 4.2. Passive Separation of Blood Pathogens

In addition to the inherent components of blood and other cells, pathogens are an important class of bioparticles in blood samples. Therefore, we present passive separation methods for the detection of pathogens in blood components using microfluidic devices.

**Figure 3:**
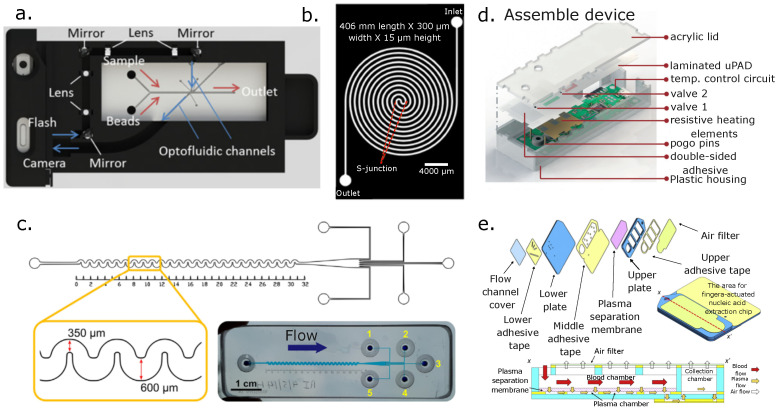
Passive and inertial microfluidic devices used to isolate pathogens from human blood. (**a**) Microfluidic device that can be coupled with a smartphone camera (reprinted from [83] with permission from Elsevier, copyright (2014)). (**b**) Template of a spiral microchip (reprinted from [85] with permission from Oxford University Press US, copyright (2021)). (**c**) Drawing in AutoCAD with microchannel dimensions and photographs of the device (reprinted from [86] with permission from Wiley Online Library, copyright (2021)). (**d**) A 3D illustration of the microRAAD for HIV testing (reproduced from [87] with permission from the Royal Society of Chemistry). (**e**) Schematic of the working principle of the plasma separation (reprinted from [88] with permission from Elsevier, copyright (2020)).

Figure 3 shows different devices for inertial and passive techniques for the separation and detection of pathogens. Stemple et al. designed a prototype of a microfluidic chip coupled to a smartphone camera to detect infectious pathogens from diluted human whole blood and a wide range of blood infections with high sensitivity [83]. The device consisted of a white LED light from a smartphone and a camera. Both are used as light sources and detectors. This light is sent to the sample mixture and the solution through optofluidic channels. The solution is inclined at 45° to capture the particles’ electromagnetic scattering or “Mie scattering”. This combination of components allowed a limit of detection between 1 pg/mL and 10 ng/mL. These results were achieved by mixing microbeads and target molecules under laminar flow in a microfluidic channel. The device presented in Figure 3 can be adapted to a cell phone through adequate coupling between mirrors and lenses. Finally, they concluded that detecting HRP-2, an antigen-specific to malaria (*Plasmodium falciparum*), from 10% blood is achievable. Further discussions about biosensors and bioelectronics on smartphones for portable biochemical detection can be found in the review by Zhang et al. [84]. These types of devices are promising for low-income scenarios where specialized photodetectors and sophisticated lab equipment are not readily available.

Batcho et al. developed a device that consists of a spiral-shaped microfluidic chip (see Figure 3b). This approach is based on inertial focusing [85], where different tests are performed using fluorescent microspheres that simulate pathogens with a fluorescent stain of *A. baumannii*. These tests presented a separation with 100% capture efficiency of the colony-forming units of the bacteria, which is in good agreement with its expected operation. This device used a design where the chip consisted of a double spiral and six loops. This architecture turned out to be quite innovative for trapping pathogens since the device was conceived for use in extreme conditions, such as in combat or war zones and in low-income countries.

Figure 3c presents a system that can pre-concentrate bacteria and later separate them from RBCs. In this work, the device consisted of a serpentine geometry with different depth dimensions (z dimensions) with inertial focusing. The authors reported separations of particles and cells with sizes from approximately 1 μm to 10 μm [86]. This device produced recoveries of up to 89% and over twice the concentration at high throughput. It also allowed for 54% separation of *Escherichia coli* and 97% for RBCs in diluted blood samples.

Phillips et al. proposed a microfluidic rapid and autonomous analysis device (microRAAD) that automatically separates and detects HIV RNA from whole blood samples using a capillary-driven lateral flow inmmunoassay [87]. This device consists of a control temperature circuit, heat-resistant silver ink, and a single-use microPAD, all contained in a plastic case as shown in Figure 3d. They incorporate vitrified amplification reagents, thermally actuated valves for fluidic control, and a temperature control circuit for low-power heating. Common HIV RNA detection methods can take 8–10 days of lab processing, whereas, with this device, the sample preparation time and detection time are significantly reduced. This device’s reagents can be dried and stored at room temperature for up to three weeks before using the device, effectively decreasing the costs for materials [87]. The authors determined that their device could detect the sample after 90 min. The device is also completely automated and has a device throughput of 2.3 × 10^7^ virus copies per milliliter of whole blood.

Kim et al. developed a microfluidic device integrated into a plasma separation chip and a nucleic acid extraction and purification chip. This system did not require an external power source to work. It relied on capillary force to infiltrate plasma and pathogens through a filtration membrane into the plasma chamber, from which it could be extracted for further testing [88]. As a model pathogen, they used *E. coli* O157:H7, which was first separated from the blood. Then, the pathogen’s DNA was purified. DNA purification was confirmed by gel electrophoresis. Their work responds to two main requirements: the need for a working device in places without in-house power sources, since it does not require electric devices, and the possibility of being extended to the separation of other strains of bacteria (see Figure 3e).

## 5. General Considerations and Parameters for Active Separations

When selecting an active separation method, which in this context means the use of applied external forces (F_ext_) in microfluidics [89], not only does the device geometry play a role, but several aspects or parameters should be considered to separate blood contents or bioparticles successfully. These parameters are the particle volume (V_p_), contrast factor, and applied external forces (F_ext_). The particle-to-separate volume is the closed system we are interested in separating, and its intrinsic properties will react to the applied force. For practical purposes, the particle volume (V_p_) usually assumes that the cells and bioparticles are perfect spheres [37]. There is no such perfect morphology for cells. Still, a spherical shape is a close approximation unless the particle presents a significant eccentricity or a highly irregular shape in which proper volume corrections should be performed. This review assumes that RBCs and the other blood components follow a spherical (V_p_) volume. However, it is well known that RBCs have a concave center region. Other blood cells, such as platelets, can also be irregular in shape. The importance of correcting this geometric factor depends on the specific application and field of study. For most blood separation applications, assuming a spherical bioparticle volume is reasonable. In this review, we present the main external forces used to separate blood components and their respective force equations to illustrate the importance of the contrast factor when selecting an active separation technique.

### Contrast Factor

The contrast factor is a concept that describes how different (or similar) the suspended particle of interest is with respect to its surrounding medium. This factor can be thought of as how visible the particle is to the applied external force. Ideally, a high contrast factor with the surrounding medium allows the particle to become more “visible” to the applied force, making the process more selective toward the particle(s) of interest. This, in turn facilitates particle manipulation into the intended positions inside the microfluidic device during the separation of blood components [90,91]. The contrast factor depends on the bioparticle’s intrinsic properties and the relation these properties have with respect to the properties of the medium [92,93]. Hence, the contrast factor should be carefully considered based on the target particle response to the exerted force (F_ext_) to achieve a high-purity separation outcome.

Another challenge arises when there are different types of particles in the sample with similar properties, as is the case for whole blood samples. Two or more particles of interest might not have significant size differences, making their separation more challenging. Therefore, the external force used to separate those particles should be the one that gives the highest contrast factor between those bioparticles. Exploiting differences in particle volumes, their response to exerted forces, and their intrinsic properties enhance blood separations. Highly selective labels can also improve separations at the expense of modifying the cell surface or interior biochemically, which could affect cell functionality. Alternatively or in combination, adding different separation stages and combining different hydrodynamic methods with external forces into a single integrated device can further increase the device’s throughput, efficiency, purity, and recovery yield [94]. The more complex the sample is, and the more fractions for downstream collection that are needed, the more likely it is that the device will require the integration of several methods and forces to achieve separations that can be scaled up.

## 6. Active Techniques

Here, we consider the active techniques that apply an external force as the main driving mechanism to separate blood and its components. These external forces interact with the target bioparticles, and depending on the difference in the physical properties of the mixture of bioparticles, they are resolved better or worse during the separation process [95]. For separating or sorting blood components and other bioparticles, it is important to consider that external forces must overcome the particle drag force since these separations are performed in a continuous flow. If the particle drag force is higher than the applied force, then the particles will follow their initial laminar flow streamline, and they will not be deflected or separated by the applied external force. We will describe the commonly used external forces for separating blood and its components. These methods are dielectrophoresis, magnetophoresis, and acoustophoresis. In addition, thermophoretic and optical gradient forces or optoelectronic tweezers will be briefly discussed as well [96].

### 6.1. Electric Forces: Dielectrophoresis (DEP)

**Figure 4:**
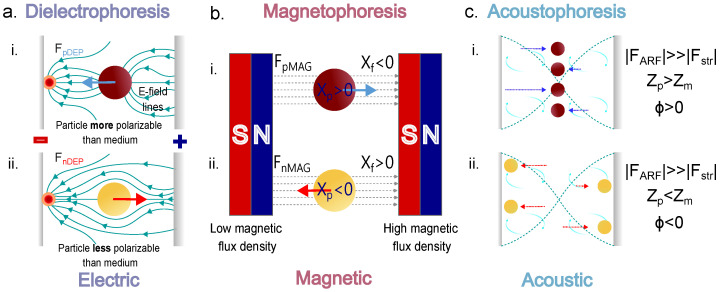
(**a**) Here, (i) and (ii) represent the high electric field points in orange (left side) and the particle of interest in red and yellow. It shows the particle displacement vector. The pDEP displacement vector is represented in (i) in blue, and nDEP is represented (ii) in red. In (**b**), the magnetophoresis concept is represented, showing the particle displacing toward (blue displacement vector) or away from the magnetic field (red displacement vector), depending on the magnetic susceptibility contrast (X_c_) between the X_p_ (particle) and X_f_ (surrounding fluid) response. (**c**) Positive acoustophoresis with a particle displacement toward the pressure node (red particles) (i) and negative acoustophoresis (ii) with a particle displacement toward the pressure anti-node (yellow particles), based on the particle acoustic contrast response.

This manipulation force is generated by applying a voltage (DC or AC) inside a microfluidic channel to create a non-uniform electric field. This non-uniform electric field can be achieved through channel geometry or different electrode configurations inside the microfluidic channel. The electric field will interact with the medium and the sample (i.e., RBCs, platelets, leukocytes, and other components), creating a polarization response in those particles toward the designed high electric field points inside the channel [97]. The polarization response from the sample depends on the electric contrast the sample has with its surrounding medium. The electric contrast, known as the Clausius-Mossotti factor, ultimately dictates how the particle will respond to the electric field’s non-uniformities inside the channel [98,99]. If the particle polarizes more than the surrounding medium, then the particle will be pulled toward the high electric field points in the microfluidic channel, and the net result of this displacement is known as a positive DEP (pDEP) response. When the particle polarizes less than its surrounding medium, it is known as a negative DEP (nDEP) response, which pushes the particle away from the high field points [100,101]. We show a diagram of these concepts in (i) and (ii) in Figure 4a, respectively. The contrast factor f_CM_ and the DEP force response F_DEP_p__ of the particle or cell to separate are defined by the following expressions:

(1) $$f_{CM}=\frac{\varepsilon_{p}^{*}-\varepsilon_{medium}^{*}}{\varepsilon_{p}^{*}+2\varepsilon_{medium}^{*}}$$

(2) FDEPp=πrp3εmediumε0Re|fCM|∇|E|2

where $\varepsilon_{p}$ and $\varepsilon_{medium}$ are the complex permittivity of the particle and the medium, respectively, *r_p_* is the particle radius or cell radius, and ${\nabla |E|}^{2}$ is the electric field strength [102].

DEP’s main advantage is that it can exploit the intrinsic electric properties of the cells and their interaction with their surrounding medium conductivity to create the desired polarization response from the particles. Thus, it can be tuned to separate them if the electric contrast between them is significant enough to be separated by adjusting the medium conductivity. In addition, when using AC voltage, adjusting the frequency such that the particles are polarized as intended provides another way to tune the separation of particles. Recently, it has been proposed that f_CM_ can be seen as the summation of the total dipole moment of a Maxwellian particle, or f_CMmacro_, and the total dipole moment of a Lorentzian particle or, f_CMmicro_. Which dipole moment contributes more significantly to the summation of the total dipole moment depends on the particle size scale [103,104]. This analysis is outside the scope of this review. Hence, for blood separations, we will assume the Maxwell-Wagner formulation derived from Maxwell’s mixture theory [105] for all the bioparticles discussed here since they follow a Maxwellian behavior due to their size scale.

One DEP caveat is that the high electric field point’s spatial extent decreases rapidly just a few microns (less than 10 μm) away from the high field point (for example, in the z direction when using co-planar electrodes). Thus, the particles need to be extremely close to those high electric field points, which presents design and experimental challenges, especially for continuous flow separations. Experimental and computer fluid dynamics experiments have been conducted to optimize the device design of dielectrophoretic devices such that the spatial electric field is further extended in the x, y, and z directions in addition to increasing the ${\left|\nabla E\right|}^{2}$ magnitude for improving the separation throughput (cells per min) of RBCs and other components [106,107]. Other electrokinetic phenomena play a role in these types of separations that can be an important resource for blood analysis. For more information on microscale nonlinear electrokinetics applied to cellular materials, including blood samples, we refer the reader to the review article by Lapizco-Encinas (2021) [108].

Another aspect that needs to be taken into account with this electrokinetic technique is that a special buffer should be used to create the electric contrast needed for separation so that the desired DEP response is attained. This means that the blood samples need to be removed from their native medium or micro-environment, causing stress that could lead to loss of viability or functionality. The buffer medium for these blood separations should not only be tailored to obtaining enough electrical contrast (right conductivity) but also be equilibrated to be isotonic so the blood particles do not experience undesired osmosis effects that can compromise their structural integrity, thus stressing them or even killing them.

In addition, other conditions should be considered based on the intended sample concentration to be separated since high blood concentrations can lead to significant cell aggregation. Hence, other agents should be added to prevent agglutination while keeping the right osmolarity conditions and medium conductivity. Buffer preparation requires several steps that take time, lead to sample loss in the washing steps, and can affect blood sample functionality. There are integrated microfluidic devices that change the blood sample medium into a DEP buffer as a stage prior to DEP separation [109]. This approach could avoid sample stress and minimize sample loss by eliminating traditional sample-washing steps.

### 6.2. Magnetic Forces: Magnetophoresis

As its name states, the magnetophoresis technique can move particles by means of magnetic forces. The key parameters that need to be considered for separating bioparticles are the cell or particle magnetic susceptibility (χ), the non-uniformity of the magnetic field, and the suspension media [110]. When the magnetic susceptibility, a measure of how prone a material is to becoming magnetized under the influence of a magnetic field, is greater than zero, the material is considered paramagnetic, and when the material susceptibility is less than zero, it is referred to as a diamagnetic material [111]. When a diamagnetic material is exposed to a magnetic field, small magnetic dipoles are created inside the bioparticle, creating a repulsive magnetic response that leads to a net particle displacement that opposes the magnetic field, known as negative magnetophoresis. In contrast, a paramagnetic particle under a magnetic field shows a weak attraction toward the field. Without a magnetic field’s influence, it behaves like a diamagnetic material since the paramagnetic effect disappears when the magnetic field is removed. Since RBCs are paramagnetic [112], they experience a net displacement toward the applied magnetic field. This response is known as positive magnetophoresis.

The magnetic susceptibility of the particles can be changed by labeling them with magnetic beads. However, particle labeling increases the cost and time of the separation process. Therefore, negative magnetophoresis tends to be more attractive since it is a label-free method for separating diamagnetic particles, such as cells in a paramagnetic medium, like ferrous solutions [113,114]. The diamagnetic particles in ferrofluids act as magnetic holes. The particle and fluid susceptibility imbalance creates a negative magnetophoretic force if the system is exposed to an external magnetic field. The negative magnetophoretic force is also proportional to the particle size. Thus, size differences between particles can also be exploited in magnetophoretic separations [115,116,117]. The magnetophoresis principle is illustrated in Figure 4b. From a particle perspective, the magnetic force F_m_p__ is expressed as follows:

(3) $$F_{m_{p}}=\left(\frac{4}{3}\pi r_{p}^{3}\right)\frac{\left(X_{p}-X_{medium}\right)}{\mu_{0}}\left(B\;\cdot \;\nabla \right)B$$

where *r_p_* is the cell or particle radius, *X_p_* is the particle susceptibility, *X_medium_* is the susceptibility of the medium, $\mu_{0}$ is the magnetic permeability in vacuum, and (B·∇)B is the magnetic field gradient of the magnetic flux density *B*. The difference between _X_p__ and *X_medium_* is known as the magnetic susceptibility contrast factor [76,112,118].

We represent the magnetophoresis concept from a particle perspective in (i) and (ii) in Figure 4b.

### 6.3. Acoustic Forces: Acoustophoresis

The acoustofluidics field exploits acoustic waves’ interactions with the sample components within a medium to separate particles such as blood cells and other bioparticles [119]. Acoustic waves emitted from a resonant device located near or within a microchannel have been used to trap, separate, and sort particles in fluids. These devices leverage the absorption of acoustic energy by the fluid to manipulate and displace the bioparticles’ positions inside a microchannel. In addition, surface acoustic waves (SAWs) [120] and bulk acoustic waves (BAWs) can be used to detect the fluid properties and to separate blood components [121]. High-frequency acoustic resonators generate the acoustic force to separate bioparticles based on their size, density, and compressibility [18].

Acoustic waves can be produced by different transducer configurations on top of piezoelectric substrates or, alternatively, by depositing piezoelectric materials on thin films [122]. Ultrasonic waves are commonly generated either by one transducer on each side of a microfluidic channel or by a single transducer with an opposing sound reflector [123]. The transducer can be in direct contact with the liquid or through a coupling layer. An external electric field polarizes piezoelectric materials which in turn produces a vibration that can be harnessed for the manipulation of particles. There are different vibration modes that depend on the piezoelectric crystal orientation to produce their characteristic signal [124,125].

The forces produced by the standing waves result in primary and secondary radiation forces directed toward the sample, thus inducing the separation of particles. The primary radiation forces are the most significant forces the particles experience. Thus, we will present applications using the particle primary radiation force in this review. To learn more about other in-depth radiation forces, publications like the tutorial review of Laurel et al. (2007) and Bruus (2012) on acoustic separation and radiation forces in small particles are good sources of information [126,127].

The wave equation for acoustics is mathematically represented by an approximate equation derived from the combination of the thermodynamic equation of state expressing pressure in terms of density, the kinematic continuity equation, and the dynamic Navier-Stokes equation [31]. The acoustic contrast factor ϕ predicts if the particle will move toward a pressure node (positive) or away from it toward an anti-node (negative) [128,129]. The acoustic contrast factor sign or particle displacement direction depends on the densities (ρ) and compressibilities (β) of the medium and the cells or particles to separate. Cells (solid) tend to move toward the standing wave pressure nodes, and bubbles (gas) tend to move toward the pressure anti-nodes. [126]. Numerical studies have been carried out to design lab-on-chip devices for separating particles in several samples, including the separation of blood components [130]. The acoustic force for a spherical particle, such as the particles contained in blood samples (e.g., RBCs, WBCs, and platelets) is expressed as follows (Equation (5)):

(4) $$\phi =\frac{5\rho_{p}-2\rho_{medium}}{2\rho_{p}+\rho_{medium}}-\frac{\beta_{p}}{\beta_{medium}}$$

(5) $$F_{Acoust_{p}}=-\left(\frac{4}{3}\pi r_{p}^{3}\right)\left(\frac{5\rho_{p}-2\rho_{medium}}{2\rho_{p}+\rho_{medium}}-\frac{\beta_{p}}{\beta_{medium}}\right)\;\cdot \;\left(\frac{\pi p_{0}^{2}\beta_{medium}}{2\lambda }\right)\sin\left(2kx\right)$$

where *r_p_* corresponds to the radius of the cell and $\beta_{p}$ and $\beta_{medium}$ correspond to the compressibility of the particle and the medium, respectively, while $\rho_{p}$ and $\rho_{medium}$ are the particle and medium densities, respectively, $p_{0}$ is the acoustic pressure, λ is the wavelength, and *k* is the wave number of the acoustic wave in the medium.

We summarize the main external forces applied for blood separations and their particles’ responses (e.g., RBCs, WBCs, and cancer cells) in the following figure (Figure 4).

### 6.4. Thermal Forces: Thermophoresis

The Soret effect or Thermophoresis is a physical phenomenon that occurs when particles suspended in a fluid are exposed to a temperature gradient. The particles will move toward the warmer or cooler regions depending on their size and charge. This effect can separate different types of bioparticles by applying a temperature gradient across a microchannel in a microfluidic device. For these types of separations, the drift velocity is the parameter that must be controlled so that the particles locate themselves in the microfluidic channel as intended for further downstream separation [131].

This relatively new technique can discriminate between particles and macromolecules (e.g., proteins and nucleic acids) in the medium. Unlike dielectrophoresis, this technique does not exhibit Joule heating issues since it does not require the application of a voltage to the media. On the other hand, acoustic waves, hydrodynamic fields, and optical properties methods rely on the basic bulk properties of the particles and lack chemical specificity. When comparing thermophoresis with magnetophoretic methods, the latter usually requires labeling cells with magnetic beads, which thermophoresis does not require. Thus far, the trapping and manipulation of yeast cells from *E. coli* and other particles with thermophoretic tweezers have been reported [132]. However, to our knowledge, no blood separation microfluidic applications have been reported to this day that separate blood and its cell components. Nevertheless, thermophoresis has the potential to be used in the near future for the separation of blood components since it has desirable features for such separation.

One of the relevant aspects of thermophoresis is its selectivity since, in principle, the temperature gradient can be carefully controlled inside microchannels due to their small confined volumes. Moreover, selectivity could be further enhanced by exploiting effects where the underlying physical mechanisms depend on specific particle-solvent interactions. Therefore, the temperature gradient ∇T across the channel should have a constant profile for effective separation, and the thermophoretic particle mobility D_T_ depends on the fluid medium and particle interactions [133]. Therefore, having a thermally responsive electrolyte is highly advantageous [134]. It has been shown that for the separation of particles, the thermophoretic force depends on the dimensionless Knudsen number. For small Knudsen numbers ($\frac{\lambda }{r\_p}$), which are characteristic of the continuum flow system found in these microscale devices, the thermophoretic force can be calculated as follows [135,136]:

(6) $$F_{T_{p}}=-24\pi \mu_{medium}^{2}r_{p}C_{s}\frac{k}{k_{p}+2k_{medium}}\frac{\nabla T}{\rho_{medium}T_{\infty }}$$

where *r_p_*, ∇T, *κ_p_*, *k_medium_*, μ_*medium*_, and *ρ_medium_* are the particle radius, temperature gradient, thermal conductivity of the particle, thermal conductivity of the medium, dynamic viscosity of the medium, and density of the medium, respectively. *C_s_* is the thermophoretic correction factor, and *T*_∞_ is the steady state temperature.

### 6.5. Optoelectronic Forces: Optoelectronic Tweezers (OETs)

Optoelectronic tweezers is a technique that combines light and electric fields obtained from the photoconductive effect of semiconductors. This technique allows the manipulation of a range of particle sizes from submicrons to tens of microns. Furthermore, OETs can only manipulate objects transparent to the applied light beam. Hence, this technique has not been demonstrated yet for hydrodynamic separations of larger particles, such as cells found in the blood. OETs have been integrated with other methods, such as electrowetting, for concentrating cells into droplets. Optical forces have been effective for separating polymers and some protein colloids, but blood separations still remain elusive, probably due to the complex nature of the optic, cell, and fluidic interactions. Despite this limitation on separating blood contents, OETs can be a valuable analytic tool for studying and analyzing cell interactions, cell patterning, and cell rotation [137,138].

## 7. Measurement and Quantification Techniques for Microfluidic Separated Blood Fractions

Quantification of the separated fractions is necessary to assess the device’s performance in terms of efficiency, purity of the fractions, cell or bioparticle throughput, sample concentrations, and more. Traditionally, sample quantification is carried out off-chip by well-established techniques such as fluorescence-activated cell sorting (FACS) or magnetic-activated cell sorting (MACS). Although FACS and MACS are robust methods, they are limited since commercially available biomarkers cannot account for the heterogeneity found in complex biological samples such as whole blood. Even the labels for some blood subpopulations can present low selectivity toward the sample of interest. Furthermore, using biomarkers requires modifying the samples with fluorescent or magnetic labels that could affect the sample functionality, and it also prevents their use for regenerative medicine applications. Finally, since these measurements are performed off-chip, valuable samples are lost when transferring the separated fractions into these platforms for quantification. Counting the collected samples with a hemocytometer is another option for some samples. This technique does not require labeling of any kind. However, it has a lower throughput when compared with FACS and MACS.

There are ongoing efforts toward integrating sensing capabilities into microfluidic devices to mitigate sample loss, save time, and reduce costs in sample quantification. Some groups have opted to integrate a customized version of FACS in their devices and even coupled their measurement platforms with microfluidic separations. Jin et al. developed a multicolor photoacoustic imaging flow cytometer in which the RBCs’ intrinsic pigment partially absorbs the incident light and converts it into an acoustic wave. With this technique, they could distinguish RBCs from melanoma cells [139]. Virtual-freezing fluorescence flow cytometry is another technique that has been explored [140], as well as label-free imaging flow cytometry [141]. Counting RBCs and WBCs from whole blood was reported with an on-chip lens-free imaging configuration [142]. More recently, an automated inertial focusing chip with two fluorescence flow measurement points (serial flow cytometry) integrated on-chip was reported by DiSalvo et al. This device was operated at extremely high velocities of more than 1 m/s, with a throughput above 100 s^−1^ and analytic yields of more than 99% [143]. Impedance measurements on-chip or impedance cytometry is another promising label-free quantification tool that counts cell events and measures the cells’ volume at low frequencies and internal cell properties at high frequencies by measuring the signal amplitude and phase [55,144]. A Dean flow fractionation device (spiral channel) with integrated impedance cytometry was capable of separating leukocytes from other blood components at a rate of 1 × 10^4–5^ cells per minute [145]. Moreover, label-free techniques that sort different blood components were also reviewed by Lu et al. (2023) [146].

## 8. Active Blood Separation Applications

### 8.1. Active Separation of Blood Cells

Since the electrical properties of RBCs are well known, numerical studies have been performed to design DEP devices for separating platelets and other blood components from RBCs. Their size differences are significantly different, thus facilitating their separation [147,148]. By using the reported dielectric properties of RBCs and platelets [36], the simulation and design of microfluidic chips to separate blood components can be carried out, ultimately exploiting their dielectric property differences or bioelectrical fingerprints, and 3D electrodes have been used to separate monocytes and macrophages [149], as well as RBCs from fixed RBCs that were treated with glutaraldehyde to induce changes in their cell membrane. The use of 3D electrodes enhances the electric field’s spatial distribution along the microchannel depth, enhancing the device’s throughput (see Figure 4a [107]. Different electrode geometries have been explored to increment the ${\left|\nabla E\right|}^{2}$ magnitude and the effective area along the x-y plane for dielectrophoretic separation and sorting [106] of blood contents. Another example of DEP separation with 3D electrodes was demonstrated using carbon electrodes. These 3D carbon electrodes separated live from dead monocytes (U937). In this case, live cells were separated from dead cells due to the differences in the dielectric properties of the two cells. Dead monocytes presented a weak nDEP or no nDEP, whereas live monocytes exhibited a stronger nDEP from 50 kHz to the crossover frequency (150 kHz) and then exhibited a strong pDEP from 300 kHz onward. Dead monocyte removal achieved approximately 90% efficiency at 1 μL/min [150]. A quite interesting device designed by Sharbati et al. also uses 3D electrodes and combines them with an array of posts to create different electric field gradients that separate different bioparticles (RBCs, T-cells, U937-MC cells, and *C. difficile*) in two steps, achieving a result of 95.5% for their multi-bioparticle separations [151]. A more recent numerical study using three-dimensional DEP effects to separate CTCs from other blood components (WBCs) demonstrated that it was capable of separating CTCs from WBCs with an efficiency of 94.3% and a fluid flow rate of ≈0.2 mL/min [152].

### 8.2. Active Separation of Blood Pathogens

A low-cost device with indium tin oxide electrodes on an acrylic substrate was used to separate plasma from whole blood (undiluted) using dielectrophoretic forces. With this device, the authors obtained a plasma purity close to 100%, and by calculating the percentage of plasma extracted compared to the plasma present in the injected whole blood sample, they obtained a plasma extraction yield of approximately 31%. Although the device’s throughput was still low, the device could be parallelized to increase the sample throughput [153]. A holography-induced DEP device was developed to sort CTCs (i.e., HT29-GFP, SW-480, and SW-620) from blood components (erythrocytes, lymphocytes, monocytes, and granulocytes). This device used interferometric phase microscopy for real-time imaging and embedded electrodes that were activated as the sorting process progressed. With this device, they successfully classified 98% of the sample, achieving a sorting accuracy of 69% [154]. A prostate-specific antigen (PSA) device used for whole blood separation first uses DEP to separate blood from plasma and then gold nanoparticles to perform an aptamer-based PSA assay. Both steps are integrated within the same chip. A detection limit of 0.25 pg/mL (see Figure 4b) [29] was achieved using this device.

One method using magnetophoresis exploits the ability to separate malaria-infected mouse red blood cells (iRBCs) from beads using V and W concave paramagnetic structures to trap iRBCs. As a proof of concept, the potential to separate iRBCs from other blood components was shown [155]. A hybrid inertial magnetophoretic device was demonstrated for separating CTCs from blood (i.e., RBCs and WBCs) in conjunction with asymmetric right-angle serpentine formations. The serpentine shape helped increase the device’s recovery rate and purity, since WBCs and CTCs are difficult to separate due to their comparable sizes. Inertial methods that rely heavily on size differences between particles struggle to separate cells of relatively similar sizes, such as WBCs and CTCs. Hence, performing the first stage that inertially separates RBCs from the rest of the sample contents (WBCs and CTCs) and later separates the remaining sample with another inertial stage to focus labeled CTCs closer to the magnet for positive magnetophoretic separation downstream helps increase the extracted CTCs’ purity and recovery rates (Figure 4c) [156]. Magnetophoretic stages have separated RBCs from WBCs in a paramagnetic mode without magnetic tagging. Deoxyhemoglobin RBCs have a higher magnetic susceptibility than other blood contents, such as WBCs and other circulating cells that behave like diamagnetic particles. Hence, an external magnetic field generated by permanent magnets creates a paramagnetic mode inside the microfluidic channels that attracts paramagnetic particles such as RBCs toward the ferromagnetic structures in the device [157].

**Figure 5:**
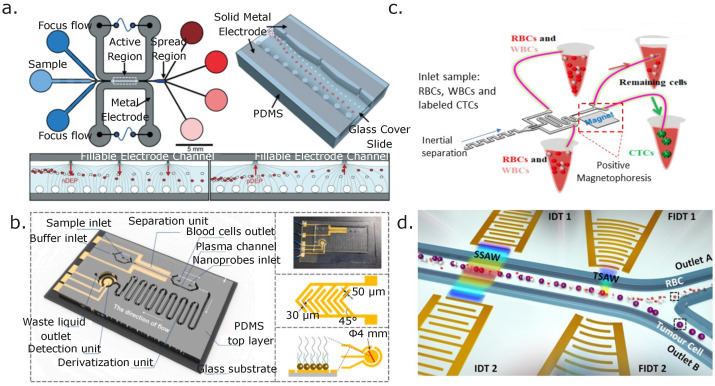
(**a**) DEP separation of RBCs from fixed RBCs (reproduced from [107] with permission from the Royal Society of Chemistry). (**b**) DEP separation of RBCs from plasma with integrated prostate-specific antigen detection (reprinted from [29] with permission from Elsevier, copyright (2022). (**c**) Inertial and magnetophoretic separation of RBCs, WBCs, and CTCs (reproduced from [156] with permission from the Multidisciplinary Digital Publishing Institute). (**d**) SAW acoustophoretic separation of glioma tumor cells from RBCs (reprinted from [161] with permission from Elsevier, copyright (2018)).

Another technique, this time a label-free one, is acoustophoresis. This technique has been used to isolate CTCs from peripheral blood mononuclear cells (PBMCs). In this work, the separation has been performed by tuning the PBMCs’ acoustic impedance to the sample medium, which differs from the CTCs’ acoustic impedance, thus enabling CTC isolation [158]. Other groups have separated CTC from spiked WBC solutions and clinical samples [159,160]. Wang et al. developed a multi-stage acoustic device for concentrating and separating the U87 glioma cancer cells with a standing surface acoustic wave (SSAW) section followed by a traveling SAW (TSAW) section. The SSAW focuses particles and cells without requiring a sheath flow. Then, the TSAW section pushes the tumor cells toward the tumor cells’ collection channel. This in turn, separates them from the RBCs’ flow streamline that is collected downstream on the opposite collection channel (Figure 5d). The authors carried out several sample separation cycles (four) and achieved a separation efficiency of 90% for the glioma cells with a viability rating of >91%. Figure 5 presents some of the aforementioned devices.

**Figure 6:**
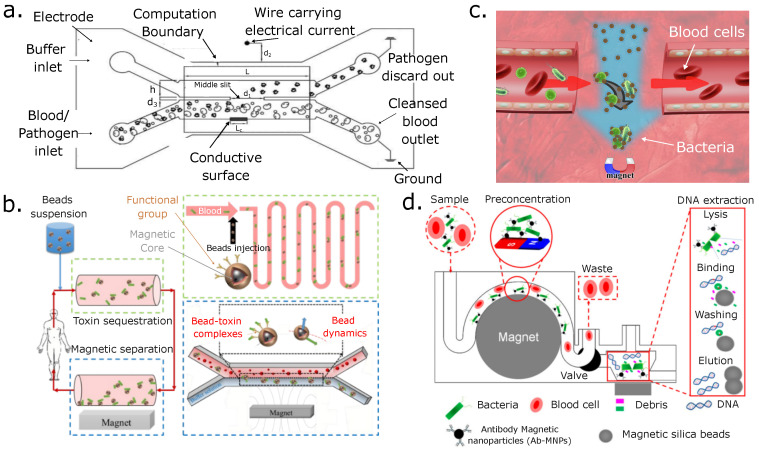
Magnetophoretic separations of pathogens in blood. (**a**) Schematic of the configuration of a magnetophoretic particle separator with two fluidic channels (reprinted from [164] with permission from Elsevier, copyright (2021)). (**b**) Device schematic about the extracorporeal blood detoxification and bead magnetophoresis (reproduced from [165] with permission from the Royal Society of Chemistry). (**c**) An illustration of the hemocompatible magnetic particles (reprinted from [162] with permission from Elsevier, copyright (2020)). (**d**) Process to separate and pre-concentrate bacteria from blood and later purify and detect the bacterial genomic DNA (reproduced from [163] with permission from the Multidisciplinary Digital Publishing Institute).

A magnetic separation application was demonstrated by Shi et al., where this group worked on the design and synthesis of a new kind of imidazolium-based ionic liquid with antibacterial activity. The polydopamine coating was utilized as hemocompatible platform to immobilize ionic liquids on Fe_3_O_4_ nanoparticles, thus forming the hemocompatible magnetic particles (Fe_3_O_4_@PDA-IL) [162]. They concluded that these particles had good hemocompatibility and were useful for removing various species of significant pathogens that can be detected in human blood. Additionally, those particles can remove bacterial endotoxins from whole-blood electrostatic and hydrophobic interactions. They also demonstrated that using IBIL-modified magnetic particles works well for removing pathogens and bacterial endotoxins from whole blood without notable effects on the blood’s complement system or coagulation of cells. Figure 6c illustrates the separation process.

In another study reported by Abafogi et al., the use of a 3D-printed design of a microfluidic prototype was demonstrated. Bacteria in whole blood were pre-concentrated, and the genomic DNA (gDNA) was purified. The separation of pathogens was achieved by magnetophoresis using antibody-conjugated magnetic nanoparticles. The absorption of lysed gDNA occurred on the surface of magnetic silica beads (MSBs), and further measurements using a polymerase chain reaction (PCR) were combined with electrophoresis and quantitative PCR [163]. The authors reported that the device could detect 10 colony-forming units per milliliter in 2.5 mL of blood, in addition to offering reduced time in the detection of these pathogens within about 50 min for the pretreatment of 2.5 mL of the spiked blood samples. A device scheme can be seen in Figure 6d. Figure 6 presents several approaches for bacterial separations using magnetophoresis.

**Figure 7:**
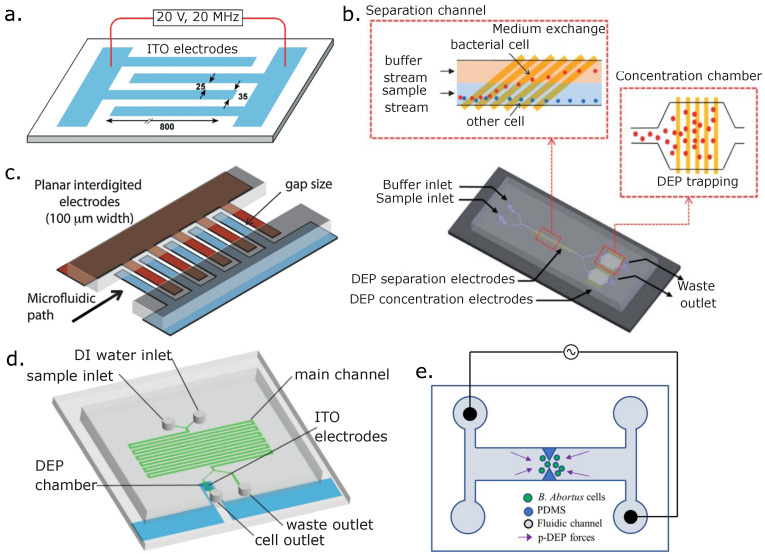
Dielectrophoretic separations of pathogens in blood. (**a**) Diagram of interdigitated ITO microelectrodes used for the universality testing (reproduced from [166], with permission from the Royal Society of Chemistry). (**b**) A microfluidic device for continuous cell separation applying DEP (reproduced from [167] with permission from the Royal Society of Chemistry). (**c**) Prototype for detecting *E. coli*, using DEP to separate the bacteria (reprinted from [169] with permission from Elsevier, copyright (2015)). (**d**) Device with an H filter and pDEP capture (reprinted from [171] with permission from AIP Publishing, copyright (2018)). (**e**) Prototype to apply eDEP and isolate *B. abortus* (reprinted from [172] with permission from AIP Publishing, copyright (2019)).

DEP has been effectively used to separate and pre-concentrate pathogens from whole blood. For example, Cai et al. proposed a technique capable of rapidly identifying pathogens from blood. In this technique, four channels are integrated and can process samples independently, identifying up to 20 different pathogens [166] (see Figure 7a). In this approach, diluted human blood was injected into the microchannels, and the pathogens were extracted from the sample using DEP and later identified by a multiplex PCR array. Notably, this device allowed the simultaneous detection of three pathogens in 3 h, including bacterial and fungal species. In addition to rapidly detecting pathogens in physiological samples, this device can potentially differentiate pathogenic microorganisms in residual water and environmental samples.

Park et al. designed a microfluidic device to continuously separate and concentrate pathogenic bacterial cells from cerebrospinal fluid and whole blood samples. This was made possible by applying DEP through high-conductivity physiological media and overcoming critical limitations in an integrated system that uptakes milliliter volumes of untreated biological samples. In this approach, cells are separated, the buffer is exchanged, and pre-concentration of the target samples up to 1 × 10^4^-fold in a volume < 1 μL is performed. This system allowed for a separation efficiency between 87.2% and 97.0% and a capture of 100% in the concentration chamber [167]. Figure 7b shows the prototype used to obtain those results.

Another application of DEP forces for trapping and isolating pathogenic microorganisms from blood cells in a complex sample is the one reported by Bisceglia et al. In this application, the sample is introduced into a microfluidic chip with an insulator-coated array of interdigitated electrodes, and after the E-field intensity and frequency bandwidth conditions inside the device are optimized, different types of target microorganisms spiked in the blood sample are separated. This work was preceded by another work from the same authors, who proposed and solved an analytical model that predicted the behavior of the fluid inside the device. Their analytical model agreed with the computational fluid dynamics simulations and their experimental results [168]. The same group also reported that they could separate *E. coli*, *S. epidermidis*, and *C. albicans* cells in a spiked blood sample using a label-free method. They captured and pre-concentrated microorganisms in a continuous flow with hypotonic buffer conditions using DEP forces [169]. Using numerical simulations, they optimized the physical parameters, such as the size of the electrode gaps, the channels’ height, applied voltage, and frequency, the dielectric constant of the particle and medium, and the flow rate. The optimization of those physical parameters satisfies fast processing and high-rate capturing of the target microorganisms in dynamic conditions with an external flow and an applied electric voltage. At a 10 MHz frequency and a flow rate of ϕ= 12.5 μL/h, pathogens are trapped and pre-concentrated around the electrode gap due to strong positive DEP (pDEP). On the other hand, WBCs are sorted through weak negative DEP (nDEP) forces and fluidic flow flushing. Using a real microfluidic device with the optimized parameters, it was possible to extract *E. coli* cells from 20× diluted spiked blood samples with a 97 % capture efficiency. Each device has four interdigitated Ti/Au electrodes of a fixed 100 μm width with various gap sizes ranging from 10 μm to 100 μm that were deposited by sputtering on a thermal SiO_2_ layer. Then, a 150 μm silicon nitride layer was deposited on top of the interdigitated electrodes to passivate the electrodes and avoid surface electrochemical reactions. Finally, a 30 μm thick dry film layer was printed with the fluidic channel shape and bonded to a glass slide employing serigraphy, thus establishing the liquid boundaries. This device is shown in Figure 7c.

Cai et al. proposed the development of an integrated microfluidic device consisting of an H-filter and two microchannels joined by a channel in the center (hence the name). H filters exploit diffusion phenomena between adjacent laminar streams to separate small particles from large particles [170]. The authors used this device principle for a desalinization stage followed by a pDEP trapping chamber to directly enrich bacterial cells from physiological samples such as cow’s milk and whole human blood [171]. Desalination of the samples occurs when electrolytes diffuse into deionized water (DI-H_2_O) before the dielectrophoretic trapping of the bacteria in a fluidic chamber. The long winding channel shown in Figure 7d exerts a prolonged osmotic and shear stress, causing hemolysis on the red blood cells but not the bacterial cells. Therefore, using permeabilizing agents that alter their dielectrophoretic behavior is unnecessary to achieve their pDEP capture. The capture efficiencies of this design range from 70.7% to 90%.

A low-cost electrodeless dielectrophoresis (eDEP) device in combination with electroosmotic (EO) and hydrodynamic pressure (HP)-driven flows was used to form a micro-vortex for rapid pre-concentration of inactivated *Brucella abortus* samples in less than 5 s [172]. This work opens the door for future manipulation and selection of bacteria that infect white blood cells in animals and humans [173]. This device could be used to enrich samples for metagenomic studies. The dielectric micro-constrictions in the microfluidic channels were fabricated using a low-cost PDMS soft lithography molding master method with a printed circuit board Cu template, providing an alternative to researchers in developing countries without advanced microfabrication facilities. To validate the experimental results obtained with the micro-chip, a multiphysics model was performed. Figure 7e shows the device’s schematics and the general working principle.

Other reviews on the use of DEP in bioseparations can offer different insights. Dash et al. presented a review of DEP focused on separating micron- and submicron-sized biological and non-biological particles, including viruses and bacteria. Moreover, they provided parameters and equations for the net DEP force, electric field, induced dipole moment, the extent of polarization as a function of the complex permittivity of the particle, the induced polarization, the split velocity between the particle and the fluid, the average temperature, and many other parameters [174]. Rahman et al. presented a review of DEP and its potential applications in medical science research, with sections focusing on the separation of viruses, bacteria, and mycoses [175]. Figure 7 presents some of the examples described for bacterial separations using dielectrophoresis.

**Figure 8:**
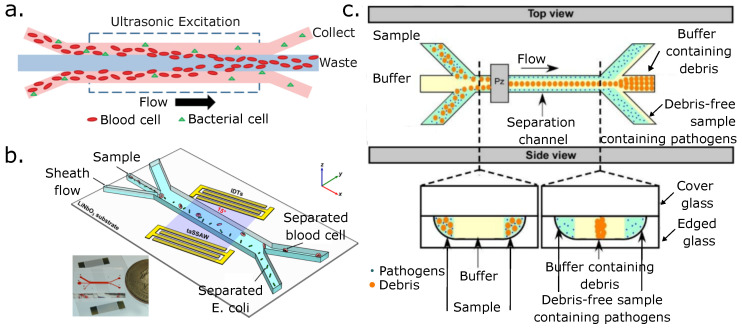
Acoustophoretic separations of pathogens in blood. (**a**) Process of acoustic separation of bacteria from blood (reproduced from ([176]) with permission from the Royal Society of Chemistry). (**b**) Illustration of acoustofluidic separation of a pathogen from human blood using taSSAW (reprinted from [177] with permission from IOP Publishing, copyright (2016)). (**c**) Illustration of the separation of large food debris particles or red blood cells from smaller intrinsic microflora (reprinted from [178] with permission from Elsevier, copyright (2016)).

Finally, acoustophoresis has also been used to separate bacteria from blood within microfluidic devices, as reported by several groups. Dow et al. separated pathogens from cells by exploiting their size difference (see Figure 8a). Once the separation process was completed, they used luminescence to detect bacteria via bacteriophages and obtain a detection rate 33 times higher than when tests were carried out without the purification step, thus achieving a yield between 40% and 60% for enriched bacteria [176]. Another application of this technique in blood-pathogen separations consisted of an acoustofluidic device that was designed to allow the separation of *E. coli* from blood using the force originating from the tilted-angle standing surface acoustic wave (taSSAW) field (see Figure 8b). Among the advantages of this device, one can highlight its (1) low cost, (2) biocompatibility, (3) flexibility, (4) easy automation, (5) higher sensitivity for the detection of pathogens from biofluids, and (6) specificity [177]. Finally, Ngamsom et al. presented a study that consisted of the separation of pathogens by acoustophoresis. (Figure 8c). This system was tested experimentally and showed the separation of *Salmonella typhimurium* with pathogen recoveries from 60% to 90% and 99.8% depletion of red blood cells after partitioning in the buffer stream. This approach is thought to be useful for preparing samples before analytical detection [178].

## 9. Discussion

Previously, in Section 2, we discussed the key figures of merit that allow us to assess a microfluidic blood separation device’s capabilities. To recapitulate, these figures of merit are the (1) input and output concentrations (cells/mL), (2) device efficiency (as a percentage), (3) purity (as a percentage), (4) flow rate (in μL/s or mL/min), and (5) throughput (number of cells/min). While reviewing recent advancements in blood separation or sorting devices, we identified that although in the majority of the cases, authors report some of these metrics, it is not the norm to report all five of these key figures of merit explicitly in their papers.

**Figure 9:**
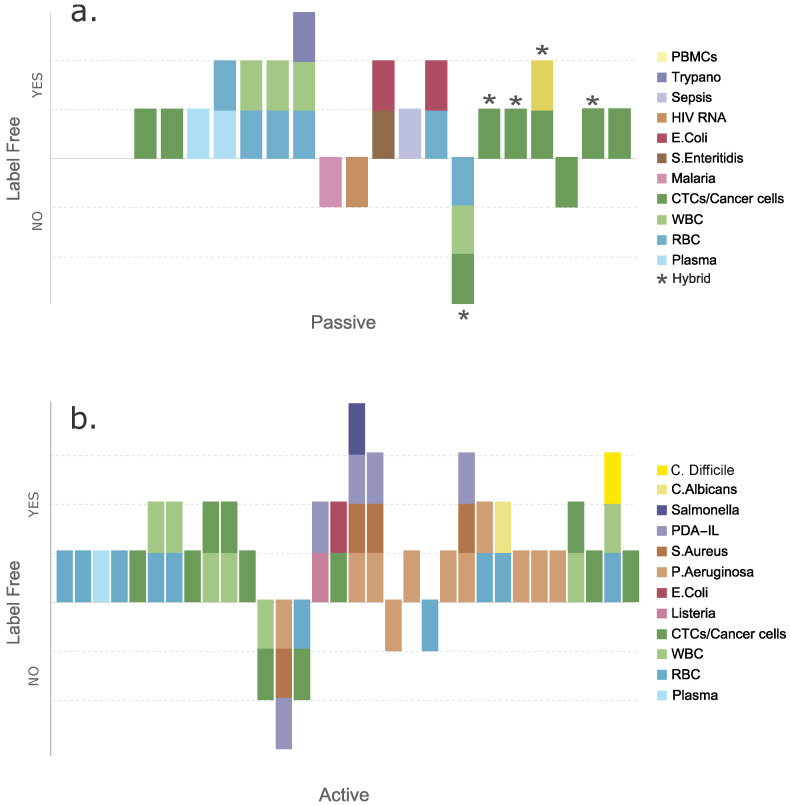
Labeled or label-free classification of (**a**) passive and hybrid as well as (**b**) active, microfluidic blood separation identifying the separated blood components.

**Figure 10:**
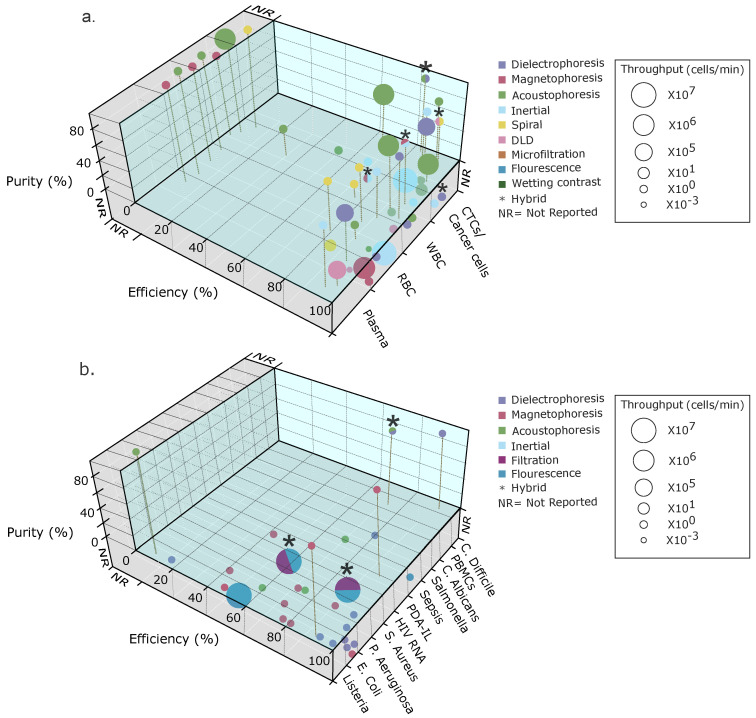
Key reported blood separation parameters. (**a**) Blood content separations and (**b**) blood separation of blood pathogens.

Figure 9 and Figure 10 summarize the reviewed blood applications with all their reported key parameters. These key parameters are used to assess the quality of the separations obtained with a device and the device’s potential for scalability. For example, for POC applications, high-quality separations are required, and a high throughput is desired for fast separations. For these reasons, we collected information from recent and novel microfluidic applications for physical separations of multiple blood contents (see Table 1 and Table 2). We identified these figures of merit for blood separations when reported to give an overview of these important metrics and identify points of improvement. In addition to these metrics, we classified the separations as label-free or labeled.

Figure 9 summarizes the methods by type of separated blood subpopulations and the number of separated components per device, and it shows whether the separation method is label-free. Figure 9a shows the evaluated passive and hybrid methods, and Figure 9b shows the active separation devices. As expected, most passive devices are label-free since they rely mostly on hydrodynamic separations (inertial) or the device geometry for separation (DLD), while one hybrid device coupled inertial separation with downstream magnetophoresis. Figure 9b presents the separations with external forces (active) and their label-free classification.

Figure 10 shows all the evaluated figures of merit for each of the reviewed blood separation devices and their separated subpopulations. In this case, we split the table information into the separation of blood intrinsic components such as RBCs, plasma, and WBCs in addition to CTCs and cancer cells (Figure 10a) as well as the separations of pathogens found in blood (Figure 10b). These three-dimensional plots allowed us to visualize the five key separation parameters we collected. The x-axis shows the type of separated cell or pathogen. The y-axis shows the reported percentage of separation efficiency, and the z-axis presents the reported purity percentage. The sample throughput is scaled to the sphere’s volume, and the color code represents each separation method. Hybrid devices were also plotted by combining the colors that represented the techniques integrated into the device in the plotted sphere, where the asterisk indicates that they are hybrid devices. These plots help to visualize which techniques present the best metrics for purity, efficiency, and sample throughput.

We caution the reader that when a sphere is located in a gray zone outside its corresponding axis (x, y, or z), it means that the authors did not explicitly report the sample’s separation figure of merit and not that the key parameter had a value of zero. To see the reported values, please refer to Table 1 and Table 2.

To summarize, acoustophoretic and inertial separations presented the highest sample throughput, with high efficiencies and sample purities for large cells such as CTCs, cancer cells, and WBCs. DLD and spiral devices fared relatively well in terms of efficiency and sample purity for separating RBCs, but they had mid-range device throughputs. The reported DEP devices have proven to be effective in separating RBCs, plasma, and cancer cells, but their separation purity and throughput are significantly lower than those for the inertial techniques. For pathogen and virus separations, DEP presents high efficiency with a low sample throughput, but since the sample purity was not reported in many instances, it is hard to establish a trend.

## 10. Conclusions and Outlook

We presented an overview of the main current blood separation techniques and what figures of merit should be reported for characterizing methods that effectively separate blood contents by microfluidic means (i.e., input and output concentrations, purity, efficiency, flow rate, and throughput). We mentioned other general considerations and parameters that should be taken into account such as the cell or particle volume, contrast factor, and external forces. The contrast factor should be carefully considered for high-efficiency and pure separations of blood components, as it affects the different exerted force responses between the components to be separated. Thus, high contrast factors will facilitate blood separation. Complex samples like whole blood will likely require different separation stages with integrated quantification on-chip for the separated fractions. Highly integrated approaches will lead to high separation efficiencies and purity as well as better separation recovery yields.

To promote the advancement of microfluidic blood separations, it is necessary to report adequate metrics or key separation figures of merit to enable comparison of the performance of the reported separations more accurately. If only a few of these separation metrics continue to be reported, then gaining a clear picture of the state of the art for blood separation techniques will remain challenging. Only one or two figures of merit will not provide the full picture of the effectiveness of an approach. There should be a consensus on explicitly reporting all five figures of merit (ideal) or at least the three main ones (efficiency, purity, and throughput) for microfluidic separation methods and devices. This information will help define the device quality and, more importantly, help the field identify critical points for improvement and establish a longer-term vision for the commercialization of these blood separation devices. Such an improvement in the reporting of these values could help these systems become an important part of the POC toolbox. Finally, we encourage the community to keep plotting these separation parameters for their devices so all of the critical metrics can be effortlessly visualized and the device performance trends can be easily identified.

## Figures and Tables

**Table 1:** Separations of blood contents.

Blood Contents to Separate	Sample Initial Concentration Volume (Cells/mL)	Separation Method	Target Sample	Separation Efficiency (%)	Purity (%)	Flow Rate (mL/min)	Cell Throughput (Cells/min)	Final Conc. (Cells/mL)	Label-Free (Y/N)	Ref.
Plasma and NCI-H1299 from RBCs	$2.2\;\times \;10^{7}$	Inertial: contraction expansions	Plasma and NCI-H1299 lung cancer cells	96 and 95	70–80	-	-	$8.7\;\times \;10^{5}$	Yes	[179]
WBCs from RBCs	500× dilution	Multidimensional double spiral	WBC (polymorphonuclear leucocytes)	>80	>85	9.2	−	12× compared with input	Yes	[72]
RBCs, WBCs, and dendritic cells	Whole blood	Inertial: spiral	Dendritic cells	77	−	−	−	−	Yes	[60]
MCF-7,Hela, and PC-3 from diluted blood	$10\;\times \;10^{7}$	Inertial: spiral with sheath flow	MFC-7 Hela PC-3	>90	-	1.4 total flowrate for separation mode	>80	-	Yes	[44]
Breast cancer cells (T47D) from blood	Diluted blood (1:50)	Inertial: cascaded sinusoidal	T47D	∼56	70.41	1	$9\;\times \;10^{7}$	−	Yes	[76]
Plasma from blood	Whole blood	Passive: microfiltration, sedimentation, and wetting contrast	Plasma	∼100	∼15 μL	-	-	-	Yes	[180]
RBCs from whole blood	Diluted whole blood ($2.25\;\times \;10^{8}$)	Dielectrophoresis	RBC	-	-	$1.68\;\times \;10^{-3}$	$1.1\;\times \;10^{5}$	−	Yes	[106]
Plasma from blood	Whole blood	Dielectrophoresis	Plasma	∼98	∼100	$0.2\;\times \;10^{-2}$	−	−	Yes	[153]
RBCs from fixed RBCs	Diluted whole blood	Dielectrophoresis	RBCs and fixed RBCs	−	−	$12\;\times \;10^{-3}$	$6.78\;\times \;10^{5}$	−	Yes	[107]
Live from dead monocytes	$3\;\times \;10^{5}$	Dielectrophoresis	Live monocytes	90	−	−	−	−	Yes	[150]
WBCs, RBCs, and CTCs	Diluted blood	Hybrid: inertial and magnetophoresis	CTCs	∼95	93	1	-	-	No	[156]
WBCs from RBCs	Whole blood	Whole blood magnetophoresis	WBCs and RBCs	89.5	−	$0.48\;\times \;10^{-3}$	−	−	Yes	[157]
Malaria-infected RBCs from magnetic beads	80% parasitemia $2.6\;\times \;10^{6}$	Magnetophoresis	−	25	−	0.4	−	−	Yes	[155]
CTC (MCF-7) from whole blood	−	Hybrid: inertial spiral, DLD, and fluourescence flow cytometry	CTCs	−	92	−	−	−	Separation yes, cytometry no	[82]
Tumor cells, RBCs, and WBCs	Diluted blood ($5\;\times \;10^{6}$) and spiked tumor cells ($10^{4}$)	Hybrid: inertial spiral and DLD	CTCs	99.9	93.59	$400\;\times \;10^{-3}$	−	−	Yes	[81]
WBCs, RBCs, and trypanosomes (*T. cyclops*)	Diluted blood sample (10x) with spiked parasite	Inertial: DLD	WBCs, RBCs, and trypanosomes	>94 (WBCs) >90 (RBCs) >80 (trypanosomes)	−	−	$1.9\;\times \;10^{-3}$	−	Yes	[73]
RBCs from whole blood	Diluted whole blood $\left(2.25\;\times \;10^{8}\right)$	DEP	RBCs	−	−	$1.68\;\times \;10^{-3}$	$1.5\;\times \;10^{5}$	−	Yes	[106]
Plasma from blood	Whole blood	DEP	Plasma	∼98	∼100	0.2×10−3	−	−	Yes	[153]
RBCs from fixed RBCs	Diluted whole blood	DEP	RBCs and fixed RBCs	−	−	$12\;\times \;10^{-3}$	$6.78\;\times \;10^{5}$	−	Yes	[107]
Monocytes (U937) and macrophages (U937)	$2.5\;\times \;10^{5}$	DEP	Monocytes	69.43	−	$1\;\times \;10^{-3}$	$10\;\times \;10^{5}$ to $10\;\times \;10^{6}$	−	Yes	[149]
WBCs from RBCs	Diluted blood	Magnetophoresis	WBCs	−	93	$20\;\times \;10^{-3}$	$10\;\times \;10^{6}$	−	No	[181]
Mononuclear cells (MNCs)	Diluted blood (20x)	Acoustophoresis	WBCs	>43 (WBCs) and >87 (MNCs)	54 (WBCs) 55 (MNCs)	$5\;\times \;10^{-3}$	$6\;\times \;10^{6}$	−	Yes	[182]
WBCs, lymphocytes, monocytes, and granulocytes	−	Acoustophoresis	−	96.8 (WBCs) 66.7 (lynphocytes) 99 (monocytes and granulocytes)	96.5 (WBCs) 71.8 (lynphocytes) 10.1 (monocytes) 98.8 (granulocytes)	0.1	$1\;\times \;10^{6}$	−	Yes	[183]
Tumor cells (U87 glioma cells) from RBCs	Diluted blood spiked with $10^{6}$ tumor cells/mL	Acoustophoresis	Glioma tumor cells	90	−	$0.3\;\times \;10^{-3}$	−	−	−	[161]
CTCs, WBCs from lysed blood	Lysed RBCs from whole blood with $1.5\;\times \;10^{6}$ WBCs spiked with CTCs	Acoustophoresis	DU145 (prostate cancer cells) and MCF7 (breast cancer cells)	42 (CTCs)	99.75 (WBC depletion efficiency)	−	−	−	No	[184]
RBCs from bacteria	Diluted blood (20%)	Acoustophoresis	Bacteria	>40 (*E. coli*) >60 (*S. aureus*) >50 (*P. aeruginosa*)	−	0.01	−	$10^{2}$–$10^{6}$ CFU/mL	No	[176]
T cells from RBCs	Diluted blood	Acoustophoresis	T cells	90.5	99	0.4	−	−	No for T cells and yes for RBCs	[185]

**Table 2:** Separations of blood pathogens.

Microorganism Separated from Blood	Sample Initial Concentration Volume (CFU/mL)	Separation Method	Separation Efficiency (%)	Purity (%)	Flow Rate (μL/min)	Cell Throughput (Cells/mL)	Label-Free (Y/N)	Ref.
Malaria	−	Passive: detecting by light	−	−	−	−	No	[83]
*Listeria monocytogenes Staphylococcus aureus* (Quickly sepsis clinical diagnostics)	from $3.2\;\times \;10^{1}$ to $4.1\;\times \;10^{6}$	Active: Magnetic separation	93.14	90	−	−	Yes	[186]
Sepsis *S. aureus*, *E. coli*, *P. aeruginosa*, and Methicillin-resistant *S. aureus*	$\;1\;\times \;10^{4}$	Electrostatic and hydrophobic interactions	from 27.3 to 80.5	−	−	−	Yes	[162]
Sepsis *S. aureus*, *P. aeruginosa*, and *E. coli*	1.42	Active: Magnetic separation and micro-Raman spectroscopy	60–70		−	−	Yes	[187]
*E. coli*	$10^{4}$	Active: dielectrophoresis	97	−	from $2.83\;\times \;10^{-3}$ to $5.67\;\times \;10^{-3}$		No	[169]
*S. enteritidis* and *E. coli*	$5\;\times \;10^{8}$ and $1\;\times \;10^{8}$	Electrophoresis	Reduce time and equipment	−	−	−	Yes	[188]
HIV RNA	Whole blood	Passive: filtration and flourescent	Depends on particle size, from 47.6% to 81.9%	$4\;\times \;10^{6}$ virus copies per mL	−	$2.3\;\times \;10^{7}$	No	[87]
*E. coli*	$10^{7}$–$10^{8}$	Electrophoresis by gel	0.3–0.6	−	−	−	Yes	[88]
RBC	−	Magnetophoresis	100	−	−	−	No	[164]
*E. coli*	$10^{1}$–$10^{6}$	Magnetophoresis	−	−	from $2\;\times \;10^{-3}$ to $20\;\times \;10^{-3}$	−	Yes	[163]
Sepsis	$10^{2}$	Fluorescence	100	−	$0.2\;\times \;10^{-3}$	−	Yes	[85]
*E. coli* and RBCs	$5\;\times \;10^{6}$ and $1\;\times \;10^{7}$	Flourescence	54 and 97	−	$0.7\;\times \;10^{-3}$	$10^{7}$	Yes	[86]
*P. aeruginosa*, *S. aureus*, and *E. coli*	$1\;\times \;10^{3}$	DEP	94.8	−	1	−	Yes	[166]
*E. coli* and RBCs	$4.4\;\times \;10^{7}$ and $1.32\;\times \;10^{8}$	Acousto-phoresis	−	96.0 and 95.85	9 and 7	−	Yes	[177]
Salmonella and RBCs	$10^{7}$	Acoustophoresis	60 and 90	−	10–30 and 10–70	−	Yes	[178]
*E. coli*	$2\;\times \;10^{7}$	DEP	milk: 90 buffer: 70.7 human blood: 80.2	−	5	−	Yes	[171]
*E. coli*	$10^{8}$	DEP	87.2	−	1.8	−	Yes	[167]
*E. coli* and *C. albicans*	$2.45\;\times \;10^{8}$	DEP (+/−)	97 (DEP+), 94 (DEP−) and 92 (DEP+), 56 (DEP−)	−	−	−	Yes	[168]

## Data Availability

No new data were created or analyzed in this study. Data sharing is not applicable to this article.
